# Abnormal Placentation After Caesarean Section: A Retrospective Study

**DOI:** 10.7759/cureus.67316

**Published:** 2024-08-20

**Authors:** Sukesh Kathpalia, Shilpa Kshirsagar, Manasvi Kulkarni, Rakshit Pandey, Jayshree Kulkarni

**Affiliations:** 1 Obstetrics and Gynaecology, Dr. D.Y. Patil Medical College, Hospital and Research Centre, Pune, IND; 2 Medicine, Dr. D.Y. Patil Medical College, Hospital and Research Centre, Pune, IND

**Keywords:** previous caesarean, scar ectopic, adherent placenta, caesarean scar, ectopic pregnancy

## Abstract

Introduction

Caesarean section (CS) is a lifesaving operation; it can have many complications in subsequent pregnancies. Since the uterine wall and cavity are not normal after CS, the implantation and subsequent trophoblastic invasion and placenta formation may be affected. This study was carried out to find out implantation and placental problems encountered in subsequent pregnancies. The spectrum includes placenta accreta, increta, and percreta and is characterized microscopically by a complete or partial absence of decidua and placental adherence to or invasion of the myometrium. The study was performed to find out the complications of CS in subsequent pregnancies and take measures to detect them early and take appropriate action.

Materials and methods

This retrospective study was carried out at Dr. D.Y. Patil Medical College and Research Centre Pimpri, Dr DY Patil Vidyapeeth, a large tertiary care centre. Many complications like placenta previa, adherent placenta, ectopic pregnancy, obstetrical hysterectomy, etc, the ones directly related to implantation and placentation, were recorded and compared with the literature.

Results and observations

The study was over a period of three years. During this period, there were 10,296 antenatal cases registered; of all the registered cases, 2,544 were cases of post-caesarean pregnancy. There were three cases of tubal ectopic pregnancy, two were diagnosed as the patients complained of amenorrhoea, spotting, and pain abdomen, confirmed on sonography and one was picked up on a routine first-trimester scan. There were two cases of scar ectopic pregnancy. Both the cases were diagnosed as threatened abortion initially and ultrasound confirmed the diagnosis; both were managed medically. Five cases of placenta previa were encountered. There were three cases of morbidly adherent placenta, and two cases underwent obstetrical hysterectomy.

Conclusion

All surgical procedures have become safe, but they all have some complications. Many complications in the next pregnancy after caesarean are life-threatening and dangerous. These complications should be detected early to prevent any catastrophic event.

## Introduction

Caesarean section (CS) has become a very safe operation due to many reasons like safe and advanced techniques of anaesthesia, improved surgical skills, better understanding of wound healing, asepsis, antibiotics, advanced neonatal care and blood transfusion facilities. Since it is a safe surgery, the doctors and patients both have become very liberal in resorting to this procedure, sometimes on trivial indications including CS on maternal request. The incidence is increasing rapidly and, in some places, it has gone beyond fifty per cent [1-3].

The acceptable incidence of CS is still not clear. At one time, a rate of 10% to 15% was considered to be acceptable; there is no empirical evidence for an optimum percentage [4]. What matters most is that all women who need caesarean sections receive them, and CS should be performed in good faith.

Though the surgery has become safe and common, it is associated with many adverse outcomes: both short term and long term. There can be many complications in subsequent pregnancies [5,6]. Rupture of the uterus, abnormal placentation, ectopic pregnancy, stillbirth, and preterm delivery can occur in future. Since the uterine wall and cavity are not normal after CS, the implantation and subsequent trophoblastic invasion and placental formation may not be normal. A retrospective study was conducted over three years to find out implantation and placental problems encountered in subsequent pregnancies after CS.

## Materials and methods

This retrospective study was conducted at Dr. D.Y. Patil Medical College and Research Centre Pimpri, Dr DY Patil Vidyapeeth, a large tertiary care centre. Institutional ethical waiver was obtained as per letter no I.E.S.C./W/51/2024 dated 30 May 2024. Most of the patients were booked but there were some cases who were admitted at the last moment or were referred from peripheral places as the place of study is a tertiary care centre. The complications that were directly related to implantation and placentation were recorded. Their management and outcomes were studied in detail and these observations were compared with the literature available. The complications that were due to abnormal implantation or placentation were included in the study. This was purely a retrospective study and the cases where there was a problem of implantation and subsequent development of trophoblast or placenta were included, whereas all other cases were excluded. The complications which were studied included placenta previa, adherent placenta, ectopic pregnancy, obstetrical hysterectomy, blood transfusion and others if any.

## Results

The study was conducted from April 2021 to March 2024; during this period there were 10,296 antenatal cases registered. Of all the registered cases, 2544 were cases of post-caesarean pregnancy, 1,836 had undergone one CS and 708 had undergone two or more CS in the past. The indications were varied; 1257 had undergone emergency CS and 955 had undergone elective CS, the rest 332 were not able to explain the type of CS. All these cases of previous CS were followed up to delivery and discharge from the hospital and the outcomes were noted. All cases encountered during the study period of three years were included. Table 1 details complications related to abnormal implantation or placentation encountered during the period of study.

**Table 1:** Summary and Demographic Details

S No	Complication	Number	Parity (P)	Mean Age
1	Ectopic Pregnancy	3	P2, P2, P2	27.3
2	Scar Ectopic	2	P2, P3	26
3	Placenta Praevia	5	P2 - 3, P2 - 2	27.2
4	Adherent Placenta	3	P2, P2 -2	27.6
5	Hysterectomy	2	P3 -2	31.5

Ectopic (tubal) pregnancy

**Figure 1:**
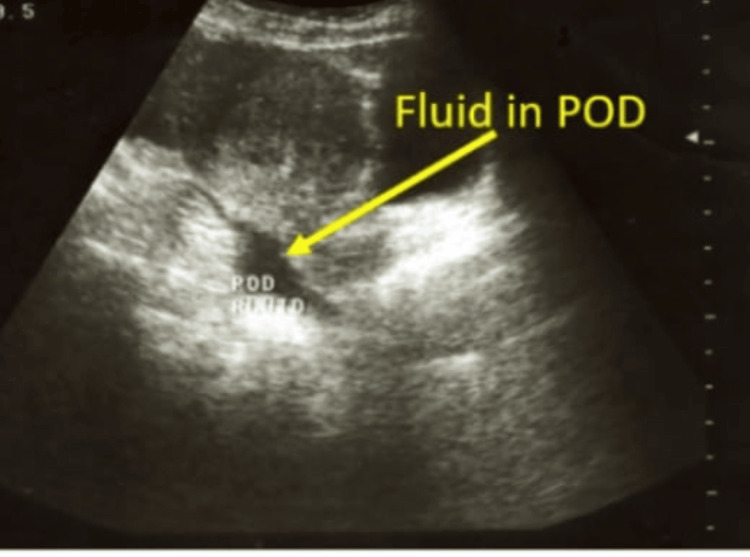
Tubal pregnancy seen on transvaginal sonography POD:  Pouch of Douglas

**Figure 2:**
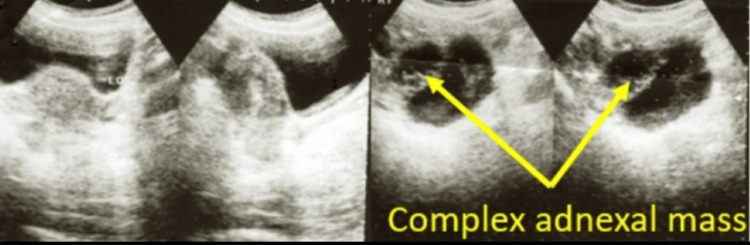
Tubal pregnancy seen on transvaginal sonography

There were three cases of tubal ectopic pregnancy; two were diagnosed as the patients complained of amenorrhoea, spotting and pain in the abdomen, confirmed on sonography (Figures 1, 2) and one was picked up on a routine first-trimester scan. One was managed laparoscopically, one was managed by laparotomy and one was managed medically by a single dose of 50 mg methotrexate. No blood transfusion was required and all three made uneventful recovery. All three cases had undergone one previous CS each.

Scar ectopic pregnancy

**Figure 3:**
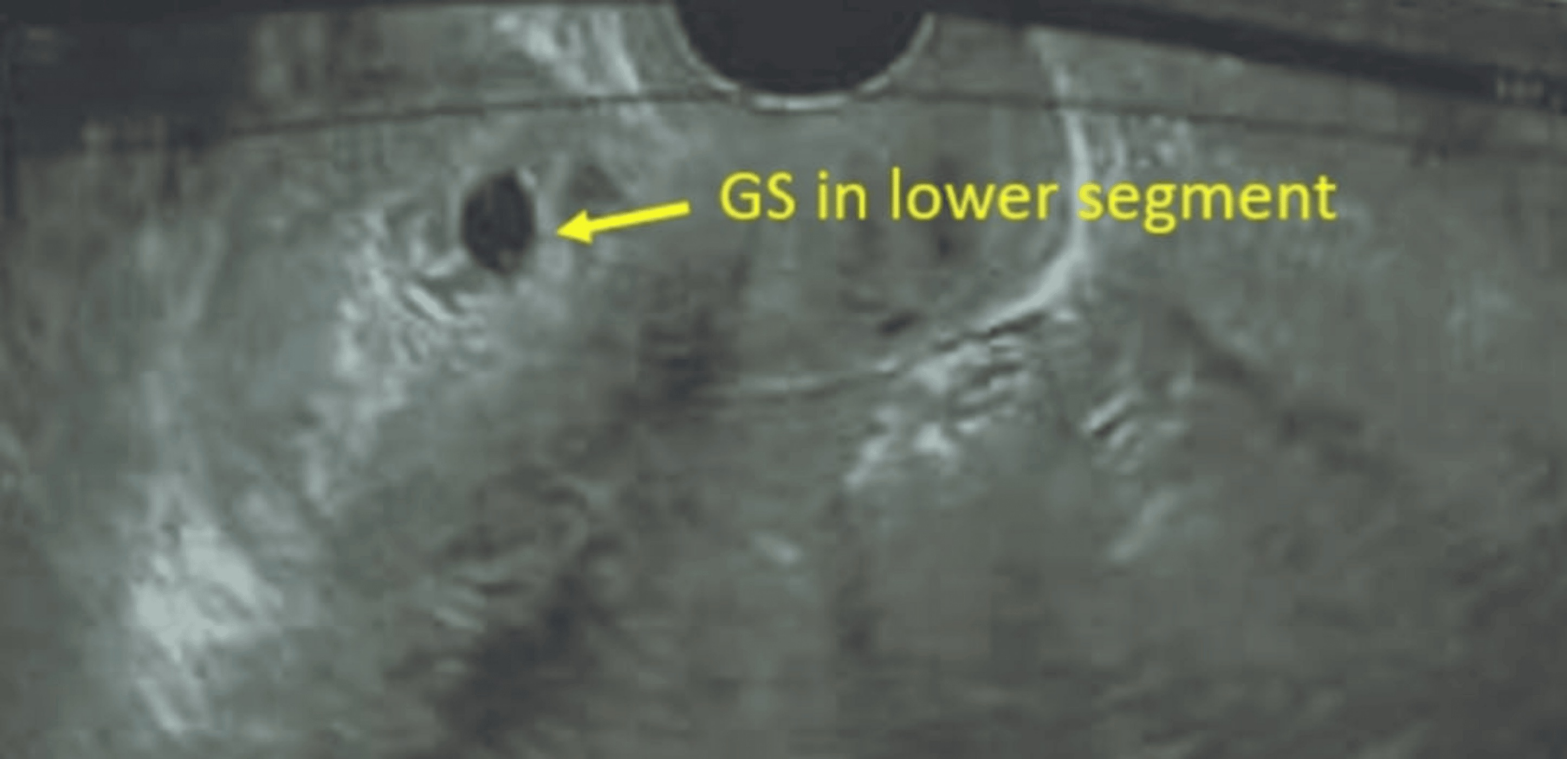
Gestational sac in the lower segment GS: Gestational sac

**Figure 4:**
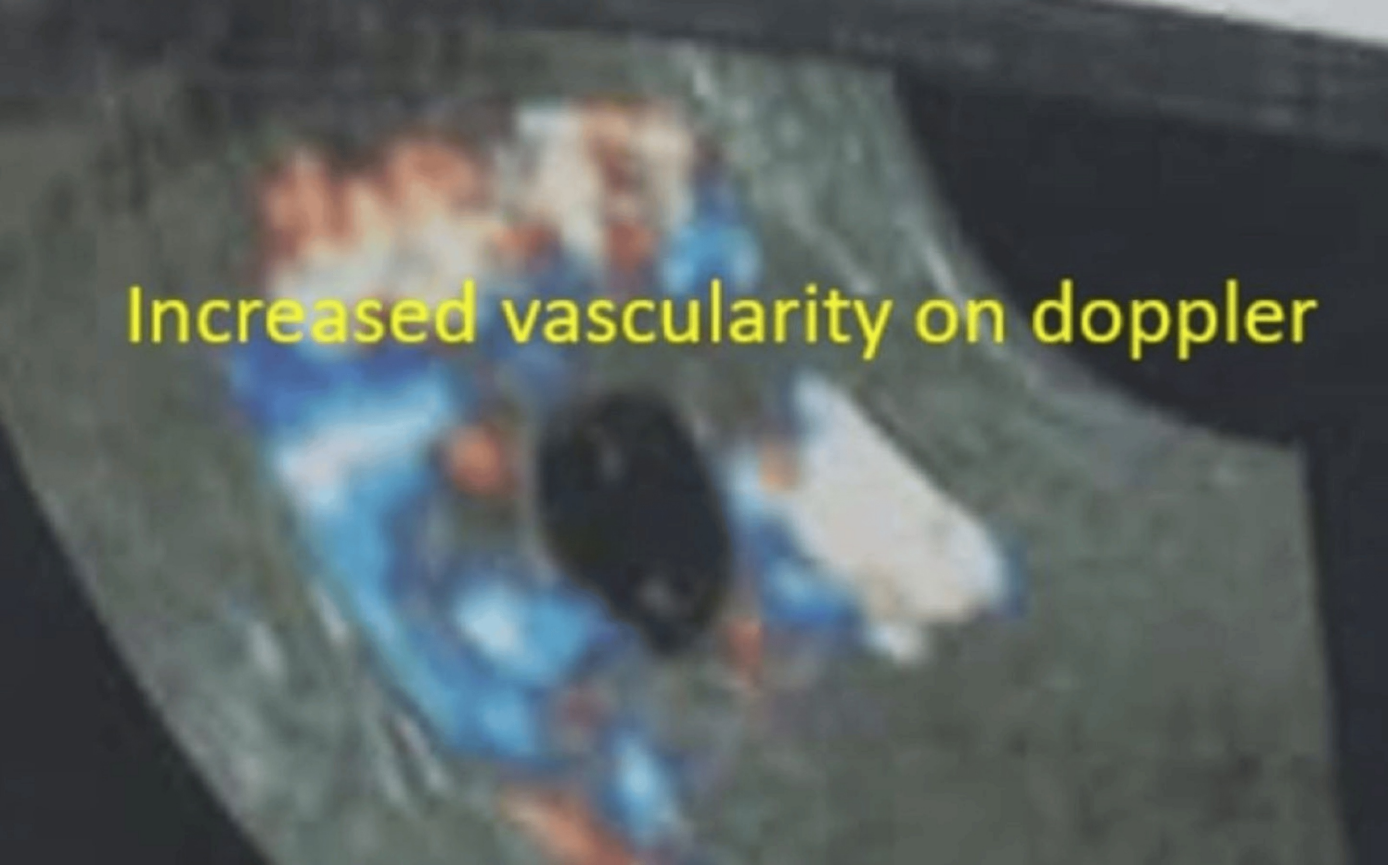
Scar ectopic pregnancy with surrounding increased vascularity

There were two cases of scar ectopic pregnancy (incidence 1/5148); both cases were diagnosed as threatened abortion initially and ultrasound confirmed the diagnosis as the gestational sac (GS) was in the lower uterine segment at the site of the previous CS scar along with increased vascularity (Figures 3, 4). Both cases did not have foetal cardiac activity and they were managed medically by injection of 50 mg methotrexate. Both cases responded well to a single dose and did not require repeated injections. One case had undergone previous two CSs and one had undergone only one section in the past.

Placenta previa

**Figure 5:**
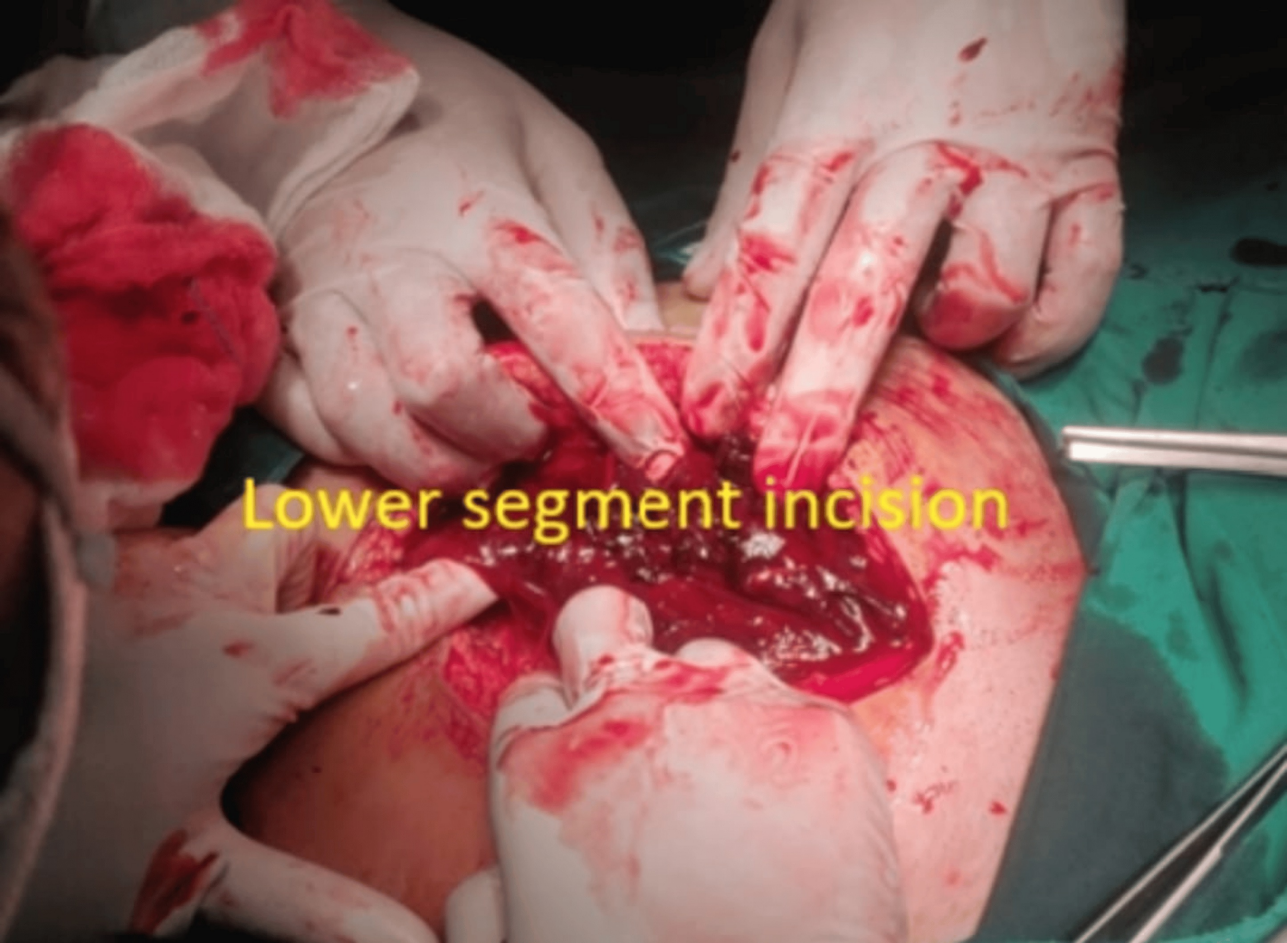
Placenta previa bleeding at the time of incision

**Figure 6:**
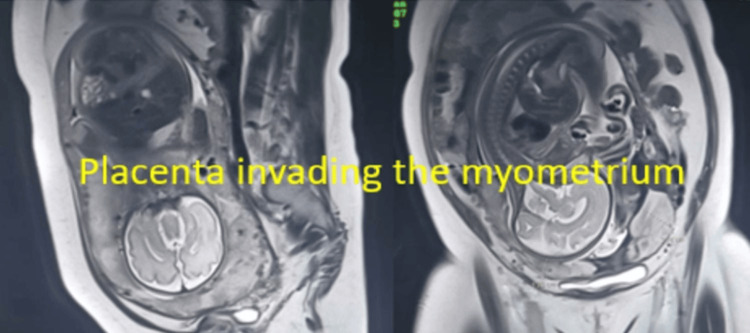
Anterior adherent placenta previa on MRI

**Figure 7:**
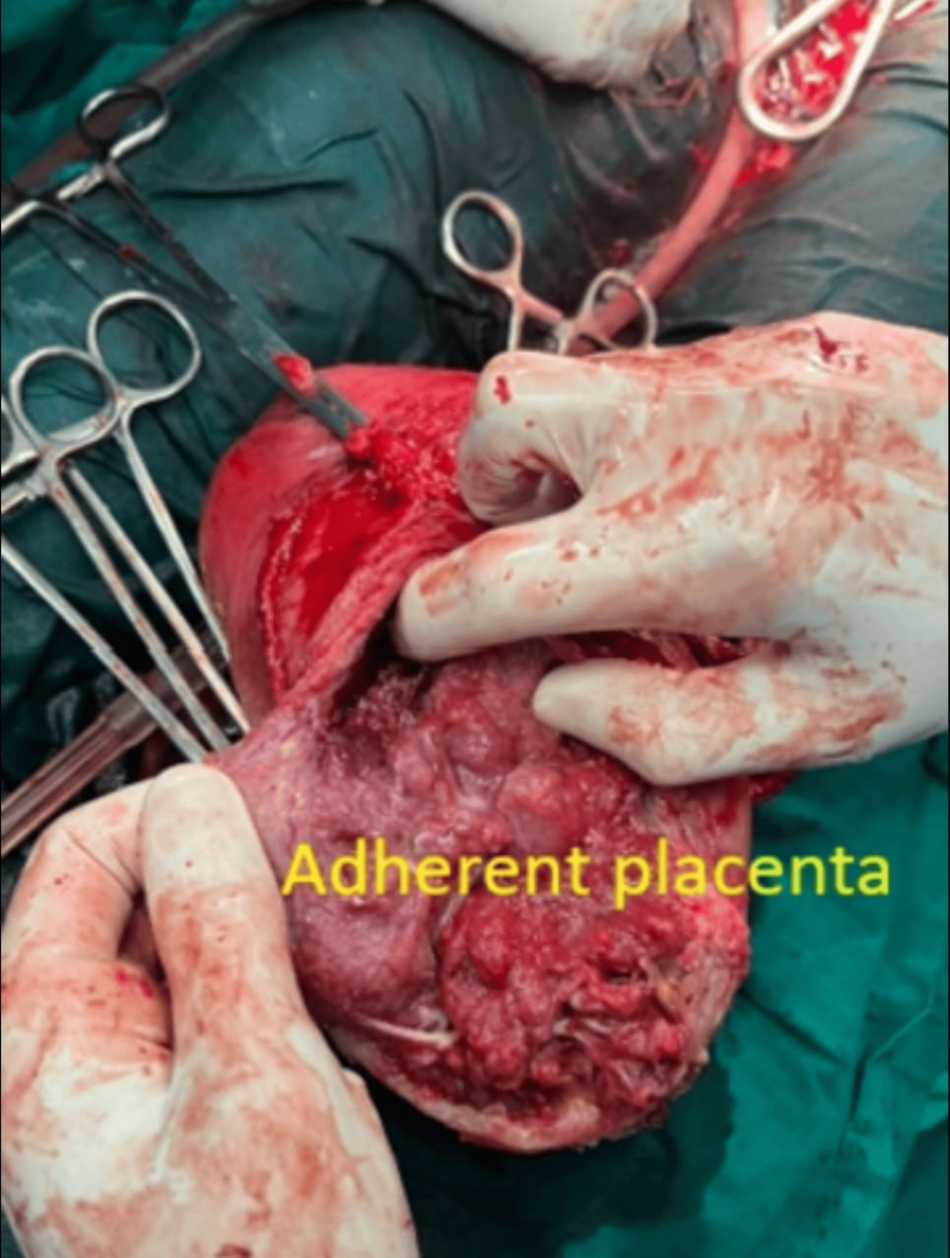
Hysterectomy specimen showing morbidly adherent placenta previa

**Figure 8:**
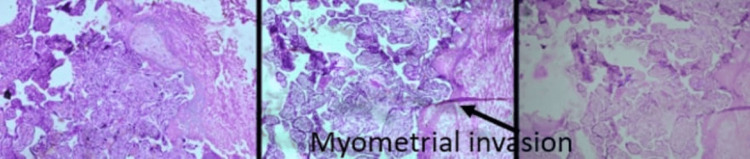
Histopathology showing morbidly adherent placenta invading the myometrium (hematoxylin and eosin staining) 10 X magnification

Five cases of placenta previa with previous CS were encountered during the study period. Four were anterior placenta previa, and one was posterior. Out of four cases of anterior placenta previa, two were not adherent and other two were adherent. The anterior placenta previa were not adherent and the posterior placenta previa had no difficulty in the repeat section except that bleeding was excessive from the beginning (Figure 5) as it happens in placenta previa cases, especially the anterior placenta previa. Of the two cases of adherent placenta previa as shown in Figure 6, one had undergone one CS in the past without any living issue, she was managed with repeat section and the adherent placenta was removed with difficulty. The second case of adherent placenta previa had undergone two previous CS and had two living children. She was counselled for hysterectomy and she agreed. In this case, the upper segment section was performed followed by an obstetrical hysterectomy (Figure 7). This case had profuse bleeding and was transfused five pints of packed blood cells. Histopathology examination (HPE) confirmed adherent placenta invading the myometrium (Figure 8); villi were observed within the myometrium.

Morbidly adherent placenta

**Figure 9:**
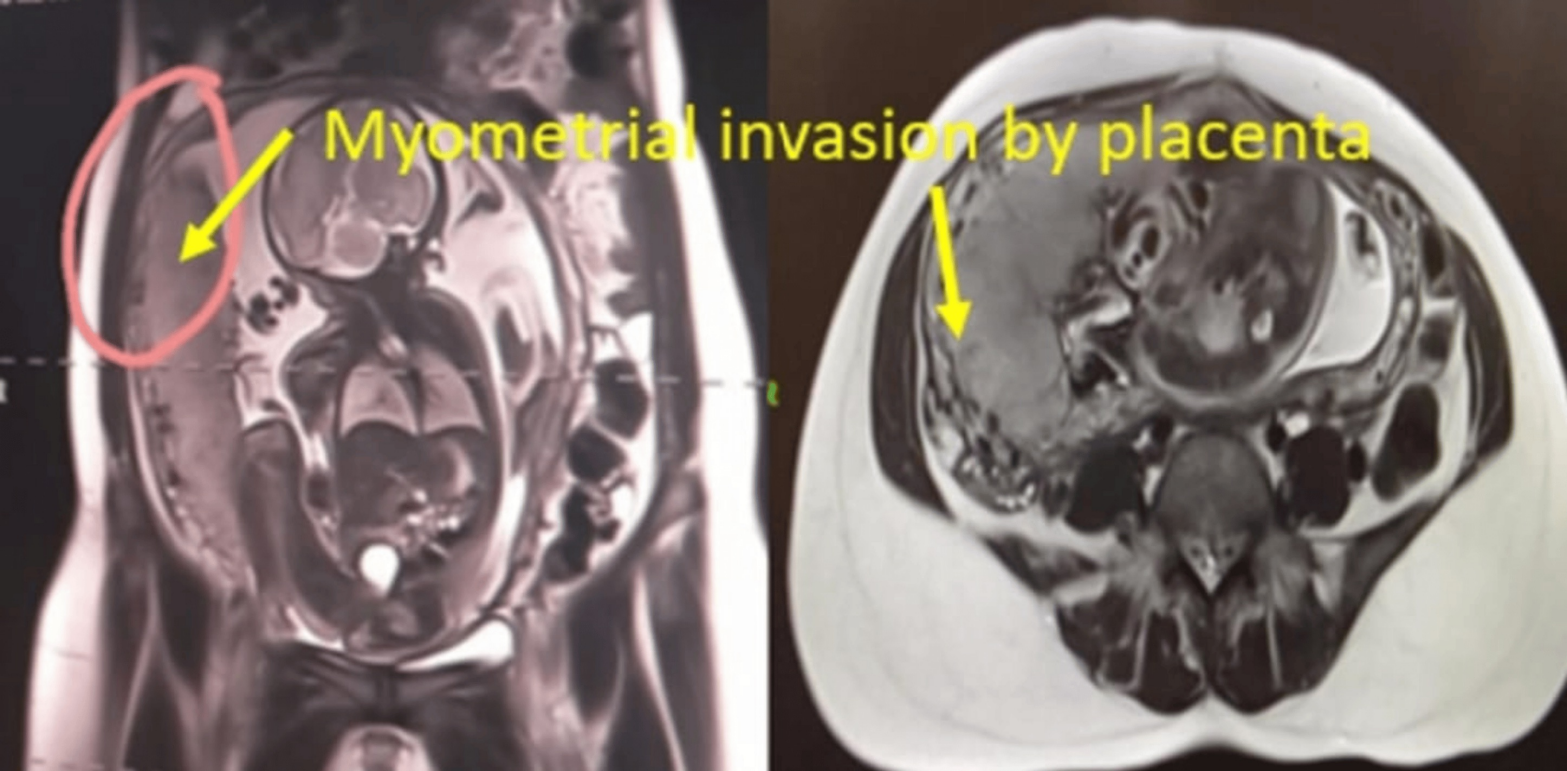
Placental adherence and invasion in the upper segment on MRI

**Figure 10:**
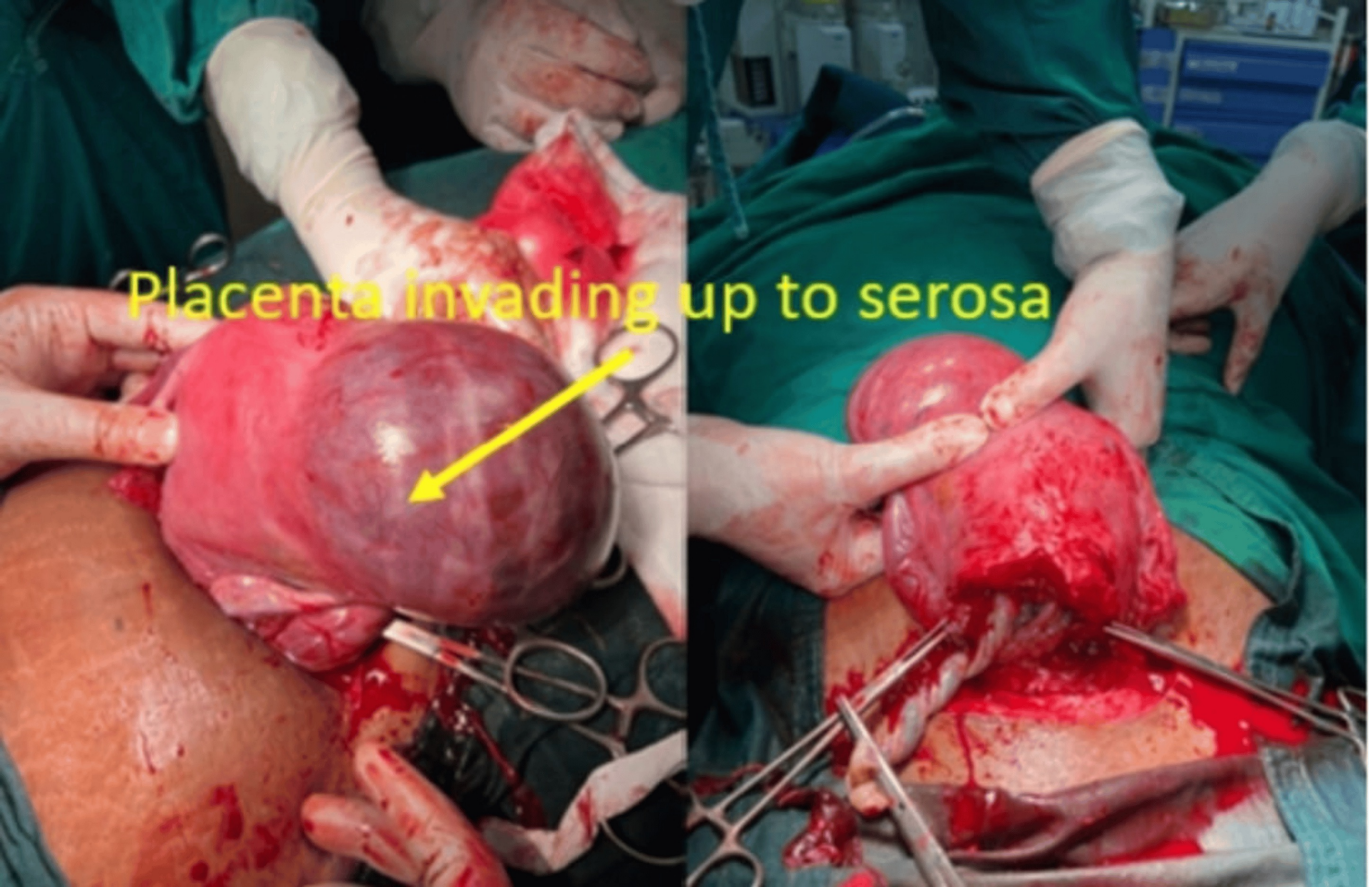
Placenta protruding from serosa

**Figure 11:**
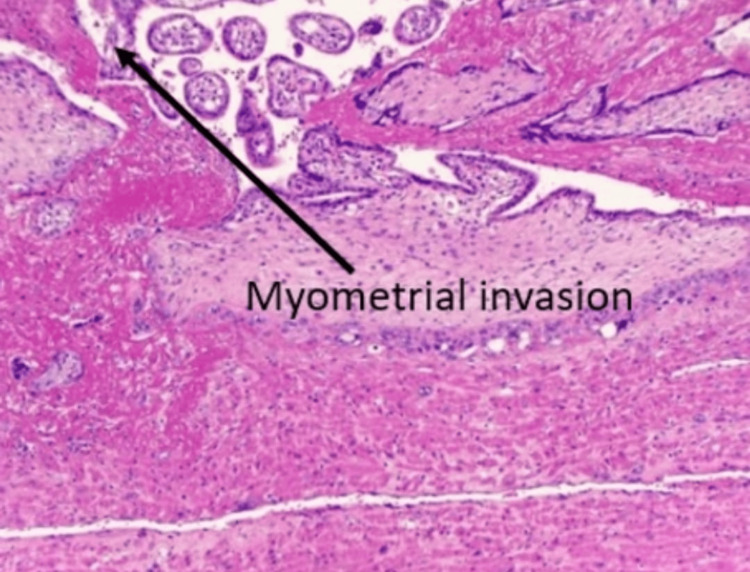
Histopathology confirmed placental adherence and invasion (hematoxylin and eosin staining) 10 X magnification

There were three cases of the morbidly adherent placenta (incidence 0.03%); two were in the lower segment as mentioned in the preceding paragraph. The third case had an adherent placenta but in the upper segment which was confirmed antenatally on MRI (Figure 9). She had her first normal delivery and had undergone a section in her second pregnancy. This patient was counselled for an obstetrical hysterectomy. She underwent a lower segment section followed by an obstetrical hysterectomy. Figure 10 shows an adherent placenta and protruding placenta through the serosa. HPE confirmed adherent placenta (Figure 11). She had an uneventful postoperative recovery.

Obstetrical hysterectomy

There were two cases of obstetrical hysterectomy, and both were performed as a planned procedure after counselling and obtaining consent. All cases of placenta previa were counselled for hysterectomy if required.

Blood transfusion

Blood transfusion was required only in placenta previa cases with or without morbid adhesions. Two were transfused two packed cell volumes each, one was given three and one was transfused five pints. Transfusion was done both during and after surgery.

Others

There were no other specific complications except a case of wound dehiscence which is not related to abnormal implantation or placentation.

## Discussion

CS has become a very common operation mainly because of its safety and easy availability. It is considered a safe operation and can save the life of the baby, mother or both. Like all other surgeries, this too has complications: operative, postoperative and long-term complications [2,5-7]. Complications may occur in future pregnancy and labour resulting in increased maternal and neonatal mortality and morbidity [6,8]. Long-term effects of CS such as infertility, pelvic adhesions, and pelvic pain are well known [6].

This study was carried out to find out complications arising due to abnormal implantation or abnormal placentation after previous CS. Only this aspect of the complications was studied. Ectopic pregnancy chances are increased in subsequent pregnancies. The reason for tubal ectopic pregnancy can be conjectured due to some postoperative adhesions which are known to occur after any surgery [7]. Caesarean scar pregnancy (CSP) is a kind of ectopic pregnancy in which the sac is implanted in the earlier CS scar at the anterior wall of the uterus. It was first described in 1978 and since then, the occurrence of CSP has been on the constant rise [8]. The estimated incidence of CSP is from 1:1800 to 1:2216; our incidence (1:5148) was found to be lower. It represents 6% of all ectopic pregnancies in women with at least one lower uterine segment incision. As the incidence of CS is rising the world over, hence the incidence of CSP is also likely to rise [9]. Diagnosis is based on clinical features and confirmation by imaging where the gestational sac is in the lower segment at the site of the previous scar along with increased vascularity [10,11]. CSP is not a very common clinical entity, correct diagnosis at the right time of CSP is crucial because the missed diagnosis or wrong diagnosis may result in dangerous and serious complications like haemorrhage, uterine rupture and even maternal death [12,13]. Low anterior implantation of the placenta is the most commonly seen early ultrasound sign pointing towards a diagnosis of CSP, although its predictive value and accuracy are not high. The treatment of CSP is still controversial and debatable. There are different ways to manage this condition and termination is better and preferred. It can be done by ultrasound-guided suction curettage along with tamponade by Foley’s catheter to prevent excessive haemorrhage [13]. In a study by Birch Petersen et al., multiple methods were suggested to manage CSP [12]. These were resection through transvaginal approach, laparoscopy, and uterine artery embolization [14] in combination with dilatation and curettage with and without hysteroscopy [12]. Hysterectomy too is one of the options; and medical therapy; either local or systemic has been tried [15]. Our two cases were managed by a single dose of 50 mg of methotrexate. Systemic medical therapy where foetal cardiac activity is present should be avoided. It is also mentioned that CSP has a very strong relationship with placenta accreta spectrum (PAS) in case the pregnancy continues. Both CSP and PAS have similar disease pathways. Indeed, it has been mentioned that pathologic findings were almost similar in the two situations.

CSP is likely a forerunner and has the same histopathological features as PAS; the two clinical situations are likely to be a continuum of the same situation with CSP being a diagnosis of the first and early second trimester, and PAS being found out later in pregnancy (second trimester and beyond). Like PAS, the rate of CSP is rising along with CS births and is likely to increase as the rate of CS births increases and as the diagnostic accuracy of imaging for CSP gets better. PAS and CSP; both involve the placenta attaching to or invading the myometrium, almost always in an area of scarring caused by earlier surgery. While PAS is typically a diagnosis made in the second trimester, some specialists and experts believe that ultrasound findings may be there in the first trimester, further confirming the association of PAS with CSP. Differential diagnosis is usually cervical pregnancy; in contrast to CSP, cervical pregnancy will happen in patients with or without having undergone a prior CS [11,16-20], whereas CSP will always have a history of prior one or more CSs.

Adherent placenta is a dangerous and potentially fatal condition which is more likely to develop where a woman has had a past CS; one or more. Its incidence is 0.13% after two CSs and rises markedly to 2.13% after four and then to 6.74% after six or more [20]. The incidence of adherent placenta was 0.03% in our study. Along with this, there is a similar rise of emergency hysterectomies at delivery [21,22]. Population-based incidence of PAS has been reported to be 4.8/10 000. Placenta previa and adherent placenta are more common after CS. PAS consists of placenta accreta, increta and percreta and is recognised histologically by a complete or incomplete absence of uterine decidual lining and placental adherence to or invasion of uterine musculature. It is a major cause of maternal morbidity and mortality worldwide [23,24]. Its incidence, reported mainly by tertiary centres, has recently reached as high as 17-90 per 10, 000 with the rise in the rates of CS deliveries [25,26]. Some studies have been conducted and those studies mentioned PAS rates within a narrower range, from 1.7 to 4.6 per 10,000 deliveries [27,28]. Incidence is higher in tertiary care centres as these centres manage high-risk cases.

It will be prudent to form a classification and stratification of women who are likely to develop PAS which would be an important step as it should help to recognise them during the antenatal period and to decide and optimize their management at the time of delivery [29-32] preferably at a higher centre. Women having both placenta praevia and at least one prior caesarean section have been identified to have a high-risk profile. This combination is associated with PAS in 10-60% of cases depending on the number of previous CS [26]. Placenta accreta happens when the placenta implants too deeply into the uterus. The risk for placenta accreta increases with each CS [33].

The chances of PAS increase when the placenta is implanted in the lower segment, as the uterine wall is thin; more so when the lower segment is scarred after previous CS. One case had a morbidly adherent placenta in the upper segment. How it occurred cannot be found out. Probably the reason could be that the placenta was removed manually which damaged the decidua resulting in the loss of the protective basal layer. It is a known fact that many specialists remove the placenta manually without waiting for spontaneous expulsion. It is our conjecture and there is no method to prove or disprove this hypothesis; it may just be an incidental occurrence [26,34]. It is not only at term that an adherent placenta can create problems; it is potentially dangerous even in the second trimester. Gautam et al. reported a case of placenta accreta in a mid-trimester pregnancy causing post-abortion haemorrhage. The patient had undergone three CSs in the past which had resulted in adherent placenta. The authors stress the importance of suspecting adherent placenta even in mid trimester in patients with a history of previous CS [35].

The study has some limitations as it is a retrospective study and not a comparative one. Detailed demographic data has not been included as the sole purpose of this paper was to study the complications due to abnormal implantation and placental development in cases of previous CS.

## Conclusions

Like all surgeries, CS too has some complications: during operation, after operation, late complications, and complications in the next pregnancy and delivery. The study was conducted to find out complications arising from abnormal implantation and placentation.

Implantation and subsequent growth can be affected as the uterine cavity along with its lining is not normal after CS. Most of the complications are life-threatening and dangerous but can be managed effectively, and these should be detected early to prevent catastrophic eventuality. 
